# Meta-analysis of the effects of on-farm management strategies on milk yields of dairy cattle on smallholder farms in the Tropics

**DOI:** 10.1017/S1751731120001548

**Published:** 2020-12

**Authors:** C. A. Bateki, S. van Dijk, A. Wilkes, U. Dickhoefer, R. White

**Affiliations:** 1 Chair for Animal Nutrition and Rangeland Management in the Tropics and Subtropics, University of Hohenheim, Fruwirthstraße 31, Stuttgart 70599, Germany; 2 Division for Agriculture and Rural Development, Unique Forestry and Land Use GmbH, Schnewlinstraße 10, Freiburg 79098, Germany; 3 Department of Dairy Science, Virginia Tech, 175 West Campus Drive, Blacksburg 24060, VA, USA

**Keywords:** smallholder farming, dairy management strategies, East Africa, mixed models, metadata

## Abstract

Although East Africa is home to one of the most advanced dairy industries in Sub-Saharan Africa, regional annual milk production is insufficient to meet the demand. The challenge of increasing milk yields (**MYs**) among smallholder dairy cattle farmers (**SDCFs**) has received considerable attention and resulted in the introduction of various dairy management strategies (**DMSs**). Despite adoption of these DMSs, MYs remain low on-farm and there is a large discrepancy in the efficacy of DMSs across different farms. Therefore, the present study sought to: (1) identify on-farm DMSs employed by East African SDCFs to increase MYs and (2) summarize existing literature to quantify the expected MY changes associated with these identified DMSs. Data were collected through a comprehensive literature review and in-depth semi-structured interviews with 10 experts from the East African dairy sector. Meta-analysis of the literature review data was performed by deriving four multivariate regression models (i.e. models 1 to 4) that related DMSs to expected MYs. Each model differed in the weighting strategy used (e.g. number of observations and inverse of the standard errors) and the preferred model was selected based on the root estimated error variance and concordance correlation coefficient. Nine DMSs were identified, of which only adoption of improved cattle breeds and improved feeding (i.e. increasing diet quality and quantity) consistently and significantly (*P* < 0.05) increased daily MYs across the available studies. Improved breeds alongside adequate feeding explained ≤50% of the daily MYs observed in the metadata while improved feeding explained ≤30% of the daily MYs observed across the different models. Conversely, calf suckling significantly (*P* < 0.05) reduced MYs according to model 2. Other variables including days in milk, trial length and maximum ambient temperature (used as a proxy for heat stress) contributed significantly to decreasing MYs. These variables may explain some of the heterogeneity in MY responses to DMSs reported in the literature. Our results suggest that using improved cattle breeds alongside improved feeding is the most reliable strategy to increase MYs on-farm in East Africa. Nevertheless, these DMSs should not be considered as standalone solutions but as a pool of options that should be combined depending on the resources available to the farmer to achieve a balance between using dairy cattle genetics, proper husbandry and feeding to secure higher MYs.

## Implications

Dairy experts have differing opinions on how effective different dairy management strategies are for increasing milk yields of dairy cattle on smallholder farms in the Tropics. These differences persist due to a lack of consensus regarding testing conditions for assessing the efficacy of available dairy management strategies. Quantitative estimates of how each management strategy contributes to the daily milk yields observed on-farm, while accounting for different testing conditions, are needed to reach a consensus among dairy experts. This study provides quantitative evidence of how much each dairy management strategy could contribute to cattle milk yields on-farm in tropical East Africa.

## Introduction

Dairy production plays a vital role in the lives of millions of rural, peri-urban and urban farmers in tropical Sub-Saharan Africa. Milk produced contributes to improved household nutrition and serves as a basis of income-generating activities related to milk processing, thus empowering youths and women (Ngongoni *et al.*, 2006). In addition to supporting economic diversification, dairy farming provides a means for nutrient recycling since manure produced can be used as fertilizer in mixed crop-livestock systems in Sub-Saharan Africa (Rufino *et al.*, 2009). Consequently, the role of dairy cattle farming on the continent cannot be over-emphasized.

There is a rising demand for dairy products across the Tropics, and especially in Sub-Saharan Africa (Gillah *et al.*, 2014). Within Sub-Saharan Africa, the Eastern African (**EA**) region (i.e. Eritrea, Djibouti, Ethiopia, Somalia, Uganda, Kenya, Tanzania, Burundi and Rwanda) produces over 5.0 billion liters of milk annually, making its dairy industry one of the most advanced on the continent (BLGG-Research, 2013). About 80% of the milk in EA is produced and marketed by smallholder dairy cattle farmers (**SDCFs**) (Rademaker *et al.*, 2016). These SDCFs typically own 1 to 10 dairy cows, each yielding less than 10 l of milk daily (Kahi *et al.*, 2000; Richards *et al.*, 2015). Despite having a more advanced industry than elsewhere on the continent, annual milk production does not yet meet the demand in the EA region (BLGG-Research, 2013). As the regional human population increases, incomes rise and urbanization continues, this deficit in domestic milk supply will become a food insecurity challenge. Thus, increasing milk yields (**MYs**) on smallholder farms is a priority for enhancing the well-being of consumers, producers and their families.

Any increase in the productivity of dairy cattle farms must be achieved through a corresponding improvement in husbandry by the SDCFs. Several dairy management strategies (**DMSs**) for improving dairy husbandry have been reported in the literature from the EA region. However, field surveys in the region still reveal substantial between-farm variation in MYs despite the implementation of similar DMSs (Ngongoni *et al.*, 2006; Kasulo *et al.*, 2010). Consequently, it is difficult to quantitatively establish expectations for how MYs should change when individual DMSs are implemented on-farm in EA. The inconsistency in MY responses to DMSs, as well as heterogeneous on-farm production conditions (e.g. environment, animal breeds and farmers’ specific practices), has led to divergent opinions among dairy experts in the region as to which DMS they consider to be most effective for increasing MYs on-farm (Biwott *et al.*, 1998; Gillah, *et al.*, 2014; Richards *et al.*, 2015). Thus, there is a need to integrate existing experimental data and expert opinion to generate estimates of the expected MY changes associated with applying different DMSs on-farm.

For such integration, a meta-analysis is an efficient tool that allows for the integration of numerical data from several studies to statistically estimate overarching average responses that can be expected from interventions applied (Fagard *et al.*, 1996). The results from these meta-analyses can then be compared to expert opinion from the region to establish how consistent literature data are with actual MYs on-farm.

Accordingly, the present study: (1) identified DMSs employed by SDCFs in EA to increase MYs and (2) summarized existing literature to quantify the expected MY changes associated with these DMSs identified.

## Material and methods

A two-stage methodology was employed to achieve the objectives of the present study. First, a quantitative literature review (i.e. meta-analysis) was performed to organize the available literature on DMS applied by SDFC in EA and quantify expected effects of these DMSs on MY. Second, in-depth interviews were conducted with various experts in the dairy sector in EA and the results of these interviews were compared with those from the meta-analysis.

### Quantitative literature review

#### Data collection

A comprehensive literature search was conducted using the Google Scholar, Google and Scopus search engines to identify articles, dissertations and reports that quantified changes in MY due to application of specific DMSs in the EA region. The inclusion criteria were jointly defined by two reviewers and studies were considered for inclusion into the dataset if they were (i) written in English, (ii) conducted in EA, (iii) evaluated dairy cattle and (iv) tested a DMS applied with the aim of enhancing MYs on-farm. The searches were performed by one reviewer using keywords and phrases (online Supplementary material S1) including MY, dairy technologies that increase MY; cattle husbandry practices that increase MY; dairy farming in Kenya; techniques for increasing dairy cattle MY; dairy cattle; napier grass; calliandra; leucaena and smallholders. The keywords and phrases were combined in various ways, such as ‘interventions OR target population OR outcomes’, ‘interventions AND problems targeted’, ‘interventions AND outcomes’ and ‘(interventions OR target population OR problem targeted) AND outcome’.

Following the online literature search, the bibliographies of the collected studies were examined to further identify relevant studies for subsequent evaluation and inclusion into the dataset. All search results were further screened by one researcher using the inclusion criteria described above before selection for further evaluation. A study was considered a relevant evaluation of MY if changes in MY associated with the DMS were recorded. Thus, MY changes must have been demonstrated by comparing the final results with either a control treatment from the study or MY from other studies or projects of similar design. Information on animal and environmental variables that could influence the MY performance of the cattle was also recorded for inclusion into the metadata.

#### Data cleaning

In several studies where the weather and altitude data were not given, these were obtained from other studies carried out in the same area. In some cases, it was not possible to obtain the corresponding data for the particular studies. Studies where weather and altitude data were missing or not available were not used in deriving the regression models that evaluated these parameters.

Data were screened for outliers based on visual appraisal of variable distributions and evaluation of means and SD of variables expected to be normally distributed. The standard errors (SEs) of reported MYs were collected from each study. Despite diversity in experimental designs and statistical approaches used by the various studies, no influence of experimental design or statistical approach on SE was identified. In terms of experimental design, studies used in our metadata included observational, completely randomized, randomized block and crossover designs. When ANOVA was used to compare SE values across experimental designs or statistical approaches reported in studies, these factors did not significantly affect SE. Hence, although some previous studies (Roman-Garcia *et al.*, 2016; White *et al.*, 2016; Martineau *et al.*, 2017) have adjusted SE for statistical approach or experimental design prior to meta-analysis, we did not adjust SE in the present study due to lack of evidence of statistical differences among populations. To prevent overweighting of particularly precise studies, SEs were curtailed (Roman-Garcia *et al.*, 2016). Following our previous work (Roman-Garcia *et al.*, 2016; White *et al.* 2017), we iterated through curtailing SEs at ½, ¼ and 1/8 of the mean SE. The cutoff at 1/8 of the mean SE was selected, because it resulted in only 4.7 % of the observations being adjusted, thus only the most extreme SE was moderated.

A major challenge with many meta-analyses is that numerous research articles fail to report SE and thus cannot be included in the analyses. In an attempt to overcome this challenge, Liebe and White (2018) tested the possibility of weighting studies with missing SE with the average SE of the dataset. In this evaluation, we have employed four approaches (four models) to handle missing SE data. In the first approach, model 1 was fitted without the use of any weighting. Second, model 2 was fitted using the number of observations (i.e. cows) to which each DMS was applied as the weighting factor. Model 3 was fitted using the inverse of the SE as the weighting factor, and all studies where SE was missing were not used in the model fitting process. Finally, model 4 used the inverse of the SE as weighting factor and for any studies where SE was missing; the mean SE of the dataset was used as weighting factor. Results from all four models are reported and compared.

### Model derivations

The model derivation procedure was done as described by Roman-Garcia *et al.* (2016) using the lmer package (Kuznetsova *et al.*, 2014) in R version 3.1.0. (R Core Team, 2014). All explanatory variables identified (i.e. DMSs and animal and environmental variables) were included in an initial multiple regression model. Then, variables were iteratively eliminated based on removing the highest (*P* > 0.1) *P*-value for each iteration until all variable *P*-values suggested at least a tendency (*P* < 0.1) for significance (*P* < 0.05). Once a model was identified that contained only significant variables or those with a tendency for significance, variance inflation factors (**VIFs**) for the variables were calculated. The cutoff for the VIF used for the present study was VIF > 10 (i.e. variance 10 times larger than a model with no collinearity) as suggested by Roman-Garcia *et al.* (2016). After removing variables with excessively high collinearity, variables were re-tested for significance and variables were continuously removed from the model until all *P*-values were below 0.1 (tendency) or 0.05 (significant) and VIF was under 10. When a final model was reached, dropped variables were iteratively added back into that final model to test whether any additional descriptors could be added back to the model without sacrificing significance and collinearity. For the models reported in the present study, the VIF of variables included in the final models was typically <2, suggesting collinearity among predictor variables was not a significant data challenge. Lastly, we tested for interaction effects between DMSs that were retained in the final models and also evaluated whether the study location (i.e. on-station *v*. on-farm) contributed to explaining the observed daily MYs.

### Evaluation of model performance

Similar to White *et al.* (2016) and Roman-Garcia *et al.* (2016), a random intercept effect for the studies was added to the linear fixed effects considered for each model. Consequently, we estimated the root estimated variance due to study and the root estimated variance for error and expressed both as a percentage of the overall mean MY per cow per day (i.e. response variable). Where possible (i.e. when identical observations were used for model derivation) the corrected Akaike information criterion was used to compare the models and identify the best model that explained the changes in MY associated with different DMSs as well as the animal and environmental explanatory variables. For consistency with other modeling efforts, root mean squared prediction error, its decomposition into mean and slope bias, and the concordance correlation coefficients were also reported to evaluate precision and accuracy of model predictions of MY.

### Expert interviews

In order to compare the findings of the meta-analysis of literature data with the actual situation in EA, experts working in different domains of the dairy sector were identified for participation in semi-structured interviews. Ten dairy experts (online Supplementary material S2) researching the health, nutrition, extension, agroforestry and genetics aspects of dairy farming in EA were selected to participate in semi-structured qualitative interviews. The experts were identified via their publications on dairy farming, their involvement in dairy-related projects in EA such as the East African Dairy Development project and their research experience in more than one country in the region. The experts were selected from the International Livestock Research Institute, University of Egerton, University of Nairobi, Centre for International Forestry Research and the World Agroforestry Centre.

The interviews were conducted using a short semi-structured questionnaire (online Supplementary material S3) and focused on defining the Kenyan and EA dairy farming context, identifying key challenges to increasing MY on smallholder farms and defining DMSs to help remedy the identified challenges. The interviews lasted between 60 and 75 min each, except in the case of the Egerton panel, where it lasted 120 min. Finally, the information from the questionnaire was matched to the corresponding DMS and ranked as reported during the interviews to identify which DMS is most effective for increasing MY on-farm.

## Results

### Data description

The literature search retained 36 studies for in-depth evaluation. Of these 36 studies, 11 were eliminated either because they used econometric approaches for estimating MY (*n* = 5) or did not meet the inclusion criteria (*n* = 7). The retained dataset (online Supplementary material S4) identified nine DMSs (Table 1) and six animal or environmental variables that could influence MYs. The 25 studies used in the present study reflected experiments performed in 4 EA countries (i.e. Kenya (*n* = 13), Ethiopia (*n* = 7), Tanzania (*n* = 4) and Uganda (*n* = 1)).

**Table 1:** Number of cattle milk yield responses considered for each dairy management strategy in the meta-analysis

Dairy management strategy	Number of milk yield responses
Calf suckling	4
Concentrate supplementation	94
Fodder crops use	34
Improved cattle breeds use	114
Improved feeding	45
Napier grass use	47
Water management regime	83
Internal parasite treatment	53
External parasite treatment	53

The 25 studies were conducted between 1989 and 2014 and applied 123 dietary and animal management treatments, representing a total of 2280 individual animal observations. The treatment comparisons considered in the present study were applied either on experimental stations or on-farm. All cattle breeds employed were representative of those used for dairy farming in the region. A summary of descriptive statistics for the nine identified DMSs is presented in Table 2.

**Table 2:** Explanation and descriptive statistics of variables from dairy cattle studies employed when fitting the mixed models

Variables	Explanation of variable as included in the model	Median	SD	Min	Max
Explanatory					
Management strategies					
Calf suckling	1 if managed to secure milk flow persistency, 0 otherwise	0.0	0.2	0	1
Concentrate supplementation	1 if concentrates are offered, 0 if not	1.0	0.4	0	1
Fodder crops use	1 if fodder crops were used in the feed supply, 0 if not	0.0	0.4	0	1
Improved cattle breeds use	1 if improved dairy breeds were used, 0 if not	1.0	0.3	0	1
Improved feeding	1 if improved quantity and quality feeds were provided, 0 if not	0.0	0.5	0	1
Napier grass use	1 if Napier grass was used as the main forage source, 0 if not	0.0	0.5	0	1
Water management regime	1 if providing water *ad libitum*, 0 if water provided less frequently	1.0	0.5	0	1
Internal parasite treatment	1 if treating or preventing internal parasites, 0 if not	0.0	0.5	0	1
External parasite treatment	1 if treating or preventing external parasites, 0 if not	0.0	0.5	0	1
Animal					
Parity of animal	1 if primiparous, 2 if multiparous and 3 for mixed group of cows	2.0	0.7	1	3
Days in milk (days)	Number of days during lactation that a cow has been milking	10	89.1	1	360
Environmental					
Season	1 if winter, 2 if summer and 3 if across seasons	3.0	0.8	1	3
Mean ambient temperature (°C)	Mean daily conditions in study area	23	3.7	14	30
Annual precipitation (mm)	Mean annual rainfall in study area	1048	440.0	75	1290
Altitude (m asl)	Elevation above sea level of study location	1850	770.0	15	2390
Study location	1 if on research station, 0 if on-farm	1	0.4	0	1
Response					
Milk yield (kg/day)	Milk yield obtained due to management strategies	6.9	3.1	1.0	14.7
Milk yield SE (kg)		0.3	0.5	0.1	2.7

Min = minimum value; Max = maximum value; asl = above sea level.

### Expert interviews

Prior to highlighting the DMSs that increases MY, the experts identified the main challenges faced in increasing MYs among SDCFs. These challenges were grouped under three aspects including animal genetics, dairy husbandry practices and dairy feeding, with each country in EA affected differently by these aspects. In terms of animal genetics, the experts held that improved dairy breeds are generally available in EA. Yet, limited accessibility of improved dairy breeds to SDCFs remains a challenge since improved breeds are still very expensive to purchase. Thus, the experts suggested that to increase daily MYs on smallholder farms, the selected DMSs should mainly focus on dairy husbandry and feeding practices. Specific DMSs suggested included: (1) feed conservation using hay and silage especially maize silage; (2) use of leguminous/feed/fodder crops to supplement grazing animals; (3) manure management for higher quality and quantity feeds via nutrient recycling; (4) appropriate use of feed supplements; (5) use of total mixed rations; (6) ensuring animal welfare (i.e. ensuring animal’s health, proper sanitation of animal sheds, feed and water, and comfort with respect to temperature extremes); (7) fertility management through feeding, correct identification of cows on heat and proper use of artificial insemination or breeding bulls and, to a lesser extent, (8) the use of home-made concentrate feeds.

### Models for management practices that increase milk yields

Four very similar models (Table 3) were obtained for explaining how DMSs affected daily MY reported in the metadata. The models differed from one another depending on whether weights were used or not and the type of weighting employed. Of the nine DMSs identified, only adoption of improved (i.e. cross-bred or exotic) cattle breeds and improved feeding (i.e. increasing diet quality and quantity offered and consumed by the cows) significantly (*P* < 0.05) increased MY in all models. The use of improved cattle breeds explained between 2.1 and 2.8 kg of the total daily MY observed depending on the model used. Also, the use of improving feeding explained between 0.4 and 1.9 kg of the total daily MY observed in our metadata. In two of the four models, an interaction was identified between the use of improved cattle breeds and the adoption of improved feeding strategies. By contrast, in one of the four models, calf suckling significantly (*P* < 0.05) reduced MY (Table 3). Of the six animal or environmental variables identified, days in milk, experimental trial length and maximum ambient temperature contributed significantly to decreasing MY. In terms of model fit, model 1 (i.e. fitted with no weighting) performed best for the statistics evaluated (Table 3). It is also worth noting that the study location and animal health variables were not retained in any of the four models fitted in the present study due to non-significance.

**Table 3:** Models showing how different management strategies affect milk yield of dairy cows on-farm

Variable	Model 1: no weight^1^			Model 2: weight by *N*^2^			Model 3: remove missing^3^			Model 4: replace missing^4^		
	Est	SE	*P*-values	Est	SE	*P*-values	Est	SE	*P*-values	Est	SE	*P*-values
Intercept	7.91	1.31	<0.001	6.85	0.86	<0.001	7.57	1.46	<0.001	7.79	1.41	<0.001
Days in milk				−0.02	0.00	<0.001						
Calf suckling				−2.06	0.60	<0.001						
Trial length	−0.01	0.00	0.004				−0.01	0.00	0.056	−0.01	0.00	0.020
Improved cattle breeds	2.79	0.54	<0.001	2.11	0.36	<0.001	2.43	0.63	<0.001	2.53	0.52	<0.001
Improved feeding	0.40	0.73	<0.001	1.25	0.46	0.007	1.89	0.34	<0.001	0.06	0.84	<0.001
Maximum temperature	−0.13	0.04	<0.001				−0.12	0.05	<0.001	−0.11	0.04	0.010
Imp. catt. breeds × Imp. feeding^5^	1.48	0.78	0.06							1.94	0.88	0.03
Fit statistics												
*N*	102			119			92			102		
Observed mean	6.37			6.97			6.52			6.37		
Predicted mean	6.37			9.95			6.45			6.31		
RMSE, % mean	15.82			17.2			16.4			16.20		
Mean bias, % MSE	0.00			0.02			0.42			0.38		
Slope bias, % MSE	0.48			1.07			0.72			0.03		
RSR	0.81			1.08			0.83			0.80		
CCC	0.93			0.92			0.93			0.93		
	2.19			3.34			2.33			2.19		
	1.13			5.23			2.56			2.49		
AICc	390			549			368			399		

Est = estimate; *N* = number of daily milk yield observations considered to fit the model; MSE = mean squared error; RSR = root mean squared error divided by population SD; CCC = concordance correlation coefficient; 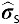 = square root of the estimated study variance; 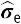 = square root of the residual variance; AICc = corrected Akaike information criterion.

1 Fitted using no weighting.

2 Fitted using weighting based on the number of observations for each management practice.

3 Using weighting based on 1/SE, and all observation without the SE excluded.

4 Using weighting based on 1/SE with mean SE used for all observations with missing SE.

5 Interaction effect between improved cattle breeds and improved feeding.

## Discussion

Of all the DMSs identified in the present study and included in the models, the use of improved cattle breeds resulted in the largest predicted increase in MY, whereas calf suckling correlated with a decrease in MY. The animal and environmental variables (e.g. ambient temperature, days in milk and experimental trial length) retained in the models were responsible for a decrease in MY in the present study. Among the four models fitted, model 1 was the most accurate and parsimonious for explaining MY increases due to different DMS. Yet, model 1 was not directly comparable to models 2 and 3 due to the different number of observations included for its fit.

### Dairy management strategies that increase milk yields *v*. expert opinion

#### Use of improved cattle breeds

In the present study, use of improved cattle breeds *v*. use of local cattle breeds explained about 30% to 50% of the improvement in daily MY observed. However, evidence from models 1 and 4 shows that up to 30% of the daily MY observed is the result of combining the use of improved cattle breeds with improved feeding. As such, a shift from local to improved breeds with the corresponding improvements in feeding could increase MYs by up to 50% of the MYs observed on smallholder farms. This finding is supported by results from previous studies that show that local breeds (i.e. *Bos indicus*) produce less milk per lactation than the improved breeds (i.e. *Bos taurus*) (Abeygunawardena and Dematawewa, 2004; Conelly, 1998). A brief look at the history of the introduction of improved cattle breeds in EA reveals that they were introduced because of their higher genetic potential to produce milk (Conelly, 1998). Despite differences in the weighting strategy used to quantify the effects of improved cattle breeds on the daily MY observed, this DMS always contributed to increasing MYs on-farm. Thus, improved cattle breeds can increase MYs on smallholder farms if adequate feeding is provided. Moreover, all experts agreed that the use of improved cattle breeds by SDCFs allows for higher MYs than local breeds. Experts hold that despite the prevalence of improved cattle breeds in the EA region, and in Kenya especially, MYs is still low. Therefore, the way forward for increasing MYs in the region requires solutions that would build on better utilizing the improved dairy cattle breeds available.

#### Improved feeding and concentrate supplementation

Improved feeding contributed significantly to increasing MY and explained between 1% and 29% of the daily MY predicted by the four models. This is unsurprising because the nutrient and energy intakes of cattle kept by SDCF in EA rarely meet their nutritional requirements, especially for improved breeds (Bwire and Wiktorsson, 2003; Place *et al.*, 2009). This undernutrition is often due to feed scarcity (i.e. seasonal or otherwise) which limits the feeding options available to SDCF. As a result, animals are fed based on the quantity and quality of feed that is available. Feeding levels and energy intakes are the main drivers of dairy cattle performance (Allen, 2000; Bateki and Dickhoefer, 2019), before and especially during lactation when their nutritional requirements increase significantly (Butler, 2000). Thus, feeding cattle on a lower nutritional plane than their requirement could lead to negative energy and/or protein balances, resulting in lower daily MYs. All the experts agreed that poor feeding is the primary factor limiting increased daily MYs in EA. Three challenges that are associated with poor feeding include the: (1) use of poor-quality feeds, (2) insufficient availability of feedstuffs and (3) frequent non-compliance by feed manufacturers with feeding standards in the feed industry. These challenges must be addressed to enhance daily MY achieved by SDCF in EA. Options to address these challenges include conserving feed and crop residues during periods of surplus, forage treatment (e.g. using urea) to improve total tract digestibility of the forages, use of alternative feedstuffs like agro-industrial by-products and supplementation of basal diets (Nyaata *et al.*, 2000; Place *et al.*, 2009). In addition, government agencies in the region should enforce regulations that ensure all feed manufacturers meet the feed quality standards in place (BLGG Group, 2016). Perhaps, most important is the need for research to determine optimum feeding levels for the cattle kept under EA conditions, since nutrient supply-driven dairy cattle performance follows the law of diminishing returns, resulting in a waste of feed resources.

Among specific feeding practices, concentrate supplementation (**CS**) was not significant in any of the models fitted, which contradicts the findings of previous studies in the region (Bwire and Wiktorsson, 2003; Muraguri *et al.*, 2004; Rufino *et al.*, 2009), as well as the opinion of most experts that CS is an effective means to increase daily MYs on-farm. This disparity in findings could be due to several reasons including: (1) the ratio of concentrate to forage in the diet as well as the nutritional composition of the concentrate used, (2) the quality of the forages used along with the concentrate and (3) days in milk and experimental trial length. In Kenya for example, the prevailing CS rate among SDCFs is 2 kg/day (Romney *et al.*, 2000; Bwire and Wiktorsson, 2003), irrespective of the quality of forages used in the diet. Consequently, cows may still have inadequate nutrient supply, and thus CS may not contribute significantly to increasing daily MYs observed on-farm. The fact that CS did not have a significant influence on daily MY observed on smallholder farms further highlights the need to develop appropriate feeding guidelines for EA dairy farming and to help SDCFs optimize feed resource use.

#### Water management regime and calf suckling

The water management regimes applied in the metadata did not contribute significantly to daily MY observed in the present study, even though previous studies had reported increases in daily MYs in various ruminant livestock species when the watering frequency was increased (Aganga, 1992; Meyer *et al.*, 2004; Khan *et al.*, 2012). The lack of significance in the present study is probably due to the fact only one study attempted to capture the effect of this DMS by using watering frequency and watering containers to estimate water intake (Muli *et al.*, 1998). Therefore, further research is needed to elucidate how access to water can affect daily MYs observed on-farm in EA.

Results from model 2 show that calf suckling contributed significantly to decreasing daily MYs observed in our study by about 30%. Three main methods exist for calf suckling, including (1) artificial calf suckling (**ACS**), (2) multiple regimes of restricted calf suckling (**RCS**) and (3) suckling *ad libitum* (Sanh *et al.*, 1995). Only two studies in our database explored calf suckling management as a DMS for increasing MY and neither authors recorded any significant increase in daily MY observed for ACS (i.e. bucket feeding) or RCS (i.e. partial suckling). Nonetheless, an increase in MY due to RCS has been reported elsewhere (Ugarte and Preston, 1975; Alvarez *et al.*, 1980; Knowles and Edwards, 1983). Although the effects are unclear, RCS is thought to stimulate milk production and persistency and thus could increase total MY harvested per lactation (Sanh *et al.*, 1995). Further potential advantages of calf suckling reported in previous studies include bacterial inhibitors in calf saliva, lower incidence of mastitis and better udder emptying (Krohn, 2001). As such, these advantages could provide justifications for encouraging better calf suckling management among SDCF. However, the experts interviewed did not consider calf suckling as a DMS for increasing MY. Rather, it was discussed as a means to secure proper development of the next generation of cows, so that sexual maturity would be attained on time to allow for more parturitions over the animal’s lifetime and thus a higher lifetime productivity.

#### Napier grass (*Pennisetum purpureum*)

Napier grass is one of the main fodders used on smallholder dairy farms in EA (Khan *et al.*, 2014). Yet, none of our four models identified Napier grass use as a significant variable explaining the daily MYs observed in the metadata. The findings from the four models concur with expert opinion, as no expert interviewed recommended the use of Napier grass to increase MYs among SDCFs in EA. The nutritional quality of Napier grass under EA production conditions has been shown to be sufficient only to satisfy the maintenance energy requirements of dairy cattle in the region (Muinga *et al.*, 1993). However, the high biomass yield capacity of Napier grass makes it attractive, since it can serve as a good feedstuff during times of feed scarcity. Other fodder options, such as those in the genus Brachiaria, have also been promoted for their positive effects on MY (Ghimire *et al.*, 2015), but further studies are needed to generate more robust evidence.

#### Further options from expert opinions

In addition to the DMS included and considered significant by the fitted models, other practices were suggested by the experts.

#### Use of leguminous fodder crops and trees

Several experts reported the use of various leguminous fodder crops and trees (**FCTs**) to increase MYs among SDCFs in EA. Various FCT species exist in EA, including *Calliandra calothyrsus, Leucaena leucocephala, Sesbania sesban* and *Morus alba* (Franzel *et al.*, 2014). Evidence of their use among SDCFs is also documented in the literature, with *C. calothyrsus* being one of the most popular species in EA due to its introduction into the area during the mid-1990s (Franzel and Wambugu, 2007). Evidence from the literature suggests that a daily MY increase of about 0.6 to 0.8 kg can be expected per kg DM of *C. calothyrsus* foliage (Place *et al.*, 2009). This increase in MY has been attributed to the higher CP concentrations of these leguminous species compared to that of most feed resources commonly used by SDCFs, which are generally characterized by low CP concentrations (Paterson *et al.*, 1998; Roothaert *et al.*, 2003). Some studies had even investigated the effects of levels of substitution of concentrate mixtures by leguminous FCTs and reported sustained levels of daily MY when dairy meal was substituted with specific amounts of either *C. calothyrsus* or *L. leucaena* (Paterson *et al.*, 1999; Kakengi *et al.*, 2001).

However, the use of FCTs to increase MYs among SDCFs faces certain constraints. First, the high content of anti-nutritive compounds (e.g. tannins and phenols) in FCT can lead to reduced DM intake and nutrient digestibility in ruminants if over-fed (Min and Hart, 2003; Huang *et al.*, 2018). For example, Barry and Manley (1984) reported that dietary concentrations of condensed tannins of >50 g/kg DM reduced voluntary feed intake in lactating ewes and adversely affected animal performance. Second, some FCTs contain toxic amino acids, such as mimosine in *L. leucaena*, which could even be lethal to animals (Brewbaker and Hylin, 1965). Consequently, it is very important that SDCFs know and use the appropriate levels of FCTs when feeding their cattle.

#### Other feed and feeding management practices

Feed conservation by haymaking and ensiling, use of feed supplements and use of total mixed rations were also suggested as practices that would increase daily MYs among SDCFs in EA. These practices all aim to improve cows’ nutrition by ensuring adequate availability and nutritional quality of feed resources all year round. These practices correspond to the building blocks that ensure improved feeding, as discussed above, and further emphasize the role of adequate nutrition in SDCF systems. However, their effective adoption has additional requirements and is contingent on the knowledge, skills and especially labor available to the SDCFs (Ngongoni *et al.*, 2006). This highlights that if the potential for increasing MY is to be realized, DMSs should be suggested in context-specific ways that match realities faced by SDCFs in the region.

### Statistical goodness of fitted models

Of the four models fitted, only models 1 and 4 are comparable due to the similar sample size (*N*) used for their derivation, while models 2 and 3 both used different sample sizes. In the case of model 3, the smaller sample size is because samples with incomplete reporting of SEs were omitted. Model 1 was the most accurate and most parsimonious as shown by the root estimated error variance. Yet, models 1, 3 and 4 explained MY increase with very similar accuracy and precision, using a similar set of variables, suggesting that the analysis captured the actual role the identified DMSs play in increasing MY on-farm. In particular, model 3 used the smallest sample size and yet selected the same combination of DMSs as those in models 1 and 4 to explain the average daily MY changes observed in the dataset. Thus, employing animal genetics (i.e. improved cattle breeds) and proper nutrition (i.e. improved feeding) under the appropriate ambient temperature conditions would enable SDCFs in EA to achieve higher MYs.

The combination of different explanatory variables in the four models and the interaction effects identified in models 1 and 4 support the assertion that no DMS can or should be viewed as a standalone solution for increasing MY (Banerjee, 2009). Rather, as was also suggested by the experts, animal genetics, proper husbandry and feeding must be combined to increase MY among SDCFs in EA successfully. With appropriate combinations, the various DMSs identified could collectively contribute to increasing MY in economically feasible, socially inclusive and environmentally friendly ways.

### Limitations

The current study has some limitations. First, identifying all relevant studies that evaluate the effect of DMSs on MYs among SDCFs in EA is challenging. Even though we attempted to identify all relevant sources, we cannot exclude that some relevant studies may have been omitted. Second, we employed a single-screening approach when applying the inclusion criteria. Hence, an influence of some systematic errors is possibly linked to the inclusion criteria applied.

## Conclusion

In summary, different DMSs are available to SDCFs in EA for improving MYs on-farm and each DMS has different potential to contribute to total daily MY. The present study suggests that the use of improved cattle breeds and improved feeding is responsible for at least 50% of the average daily MY observed on-farm among SDCFs in the region. However, these DMSs should not be considered as standalone solutions but rather seen as a pool of options that should be combined depending on the resources available to the farmer to achieve a balance between using dairy cattle genetics, proper husbandry and feeding to secure higher MY.

## Software and data repository resources

Data and R code used for the purpose of the present study are not deposited in an official repository.

## Supplementary material

For supplementary material accompanying this paper visit https://doi.org/10.1017/S1751731120001548. click here to view supplementary material
