# Supplementary material for: Meta-analysis of the effects of on-farm management strategies on milk yields of dairy cattle on smallholder farms in the Tropics
Source: Animal. 2020 Jun 30;14(12):2619–27. doi: 10.1017/S1751731120001548 (PMC7645308; doi:10.1017/S1751731120001548)
Supplement: Supplementary file 1 [file S1751731120001548sup.zip › S1751731120001548sup001.docx]

## Meta-analysis of the effects of on-farm management strategies on milk yields of dairy cattle on smallholder farms in the Tropics

Bateki CA, van Dijk S, Wilkes A, Dickhoefer U and White R 2020. Animal: An International Journal of Animal Bioscience.

## Supplementary material S1: Search strategy for meta-analysis

Search terms

*Interventions;*

- dairy management practices,
- dairy technologies,
- best dairy cattle practices,
- dairy cattle inputs,
- dairy cattle techniques,
- dairy cattle methods,
- dairy husbandry practices,
- efficient dairy cattle practices,
- dairy cattle development,
- dairy cattle programs,
- smallholder dairy development,
- dairy extension,
- management of high producing dairy cows,
- dairy cow production,
- napier grass,
- artificial insemination,
- grade breeds,
- concentrate supplementation,
- water availability in dairying,
- improved breeds,
- dairy leguminous fodder crops,
- health management in dairy cows,
- balanced rations for dairy cows,
- proper feeding for dairy cows,
- feed supplementation in dairying,
- concentrate feeding for dairy cows,
- dairy meal,
- dairy cattle breeding,
- veterinary services to dairy cow,
- balanced feed in dairying,
- agroforestry,
- fodder crops.

*Outcomes;*

- milk yield,
- productivity,
- income,
- food security,
- milk output,
- increasing milk production,
- dairy production,
- household income dairy productivity
- cost-efficient dairying
- profitable dairying
- dairy efficiency
- technical efficiency in dairying

*Target population;*

- smallholders,
- dairy farmers,
- rural dairy farmers,
- poor dairy farmers,
- small scale dairy farmers,
- smallholder dairy farmers

*Problems targeted;*

- dairy cattle mortality
- mastitis
- dairy cattle mortality rate
- dairy cattle health
- cow health
- poor health of dairy cows
- inefficient dairying
- cost-intensive dairying
- high-cost dairying
- feed scarcity in cattle dairying
- malnutrition in dairy cows
- poor dairy cattle breeds
- water scarcity in dairying
- poor quality concentrates
- over-supplementation of dairy cows

## Supplementary material S2: List of dairy experts consulted for this meta-analysis

1. Dr. Isabelle Baltenweck (ILRI)
2. Prof. Dr. Mariana Rufino (ICRAF)
3. Dr. John Goopy (ILRI)
4. Henri Kiara (ILRI)
5. Dr. Simon Fraval (ILRI)
6. Prof. Dr. Alexander Kahi (University of Egerton)
7. Dr. Bebe Bockline (University of Egerton)
8. Dr. Mary Ambula (University of Egerton)
9. Prof. Dr. Charles Gachuiri (University of Nairobi)
10. Dr. Sammy Carsan (ICRAF)
11. Dr. Steven Franzel (ICRAF)
12. Josephine Kirui (ICRAF)
13. Dr. Asaah Ndambi (ILRI)

## Supplementary material S3: Semi-structured questionnaire used during expert interview

1. What is the definition of a smallholder dairy farmer?
2. Do you think smallholder dairy farming practices are the same/similar (how similar) across East Africa? If not the same/similar, what are the main differences?
3. What is the average Kenyan Smallholder Dairy Farmers (SDF) herd size and could you say anything about the composition (ratio of males to female)?
4. What are the main challenges to increasing milk yields amongst SDF in Kenya?
5. What dairy technologies/on-farm management practices are available for SDF to employ for increasing milk yields?
6. Do you have access to any written whether scientific or grey literature showing that this/these technologies/practices were tried and produced the suggested outcome?
